# The role of endogenous opioid neuropeptides in neurostimulation-driven analgesia

**DOI:** 10.3389/fnsys.2022.1044686

**Published:** 2022-12-14

**Authors:** Susan T. Lubejko, Robert D. Graham, Giulia Livrizzi, Robert Schaefer, Matthew R. Banghart, Meaghan C. Creed

**Affiliations:** ^1^Department of Neurobiology, School of Biological Sciences, University of California, San Diego, La Jolla, CA, United States; ^2^Department of Anesthesiology, Pain Center, School of Medicine, Washington University in St. Louis, St. Louis, MO, United States; ^3^Department of Neuroscience, Washington University in St. Louis, St. Louis, MO, United States; ^4^Department of Psychiatry, Washington University in St. Louis, St. Louis, MO, United States; ^5^Department of Biomedical Engineering, Washington University in St. Louis, St. Louis, MO, United States

**Keywords:** pain, analgesia, opioid, μ-opioid receptor, neurostimulation, neuromodulation, deep brain stimulation (DBS), spinal cord stimulation (SCS)

## Abstract

Due to the prevalence of chronic pain worldwide, there is an urgent need to improve pain management strategies. While opioid drugs have long been used to treat chronic pain, their use is severely limited by adverse effects and abuse liability. Neurostimulation techniques have emerged as a promising option for chronic pain that is refractory to other treatments. While different neurostimulation strategies have been applied to many neural structures implicated in pain processing, there is variability in efficacy between patients, underscoring the need to optimize neurostimulation techniques for use in pain management. This optimization requires a deeper understanding of the mechanisms underlying neurostimulation-induced pain relief. Here, we discuss the most commonly used neurostimulation techniques for treating chronic pain. We present evidence that neurostimulation-induced analgesia is in part driven by the release of endogenous opioids and that this endogenous opioid release is a common endpoint between different methods of neurostimulation. Finally, we introduce technological and clinical innovations that are being explored to optimize neurostimulation techniques for the treatment of pain, including multidisciplinary efforts between neuroscience research and clinical treatment that may refine the efficacy of neurostimulation based on its underlying mechanisms.

## Introduction

Over 20% of people worldwide suffer from chronic pain disorders (Goldberg and McGee, 2011). In response to an unmet need for effective pain management, opioid drugs have been widely adopted. Opioid drugs harness the body’s endogenous opioid receptors, which are dispersed throughout the central and peripheral nervous system to modulate pain perception. While prescription opioids often provide effective pain relief, they have undesirable and potentially dangerous side effects including abuse liability and respiratory depression. Their contribution to the ongoing opioid epidemic and the enormous negative impact of chronic pain underscore the need for safe and effective pain therapies (Manchikanti et al., 2012). Neurostimulation therapies are potential alternatives for managing medically refractory pain. However, these therapies are hampered by inconsistent pain relief across patients and diminishing analgesic effects over time (Kumar K. et al., 1998). To optimize these therapies and predict patient responses, we must first understand the mechanisms of action underlying their therapeutic effects. The purpose of this review is to summarize the evidence suggesting current neurostimulation therapies may provide analgesia in part by driving endogenous opioid mechanisms. We conclude by discussing opportunities for multidisciplinary research to shed new light on mechanisms of neurostimulation-induced pain relief.

### Chronic pain

Chronic pain is a condition often defined by the presence of long-standing pain that persists beyond recovery of the injured tissue. In humans, chronic pain is clinically defined as pain that persists for longer than 6 months (Russo and Brose, 1998), without regard to tissue healing. One type of severe chronic pain for which neurostimulation techniques are often used is neuropathic pain, which is defined by the International Association for the Study of Pain as “pain caused by a lesion or disease of the somatosensory system” (Jensen et al., 2011). In the United States, an estimated 20.5% of adults suffer from a chronic pain condition, with 10% experiencing high-impact chronic pain that limits work and diminishes quality of life (Yong et al., 2022). This figure is mirrored by an estimated global prevalence of chronic pain of 18% (Sá et al., 2019). Many patients experiencing chronic pain are inadequately treated, with estimates ranging from 40 to 77% depending on pain etiology and study parameters (Deandrea et al., 2008; Majedi et al., 2019). Due to its high prevalence worldwide, there is a clear and urgent need for safe and effective therapies for managing chronic pain.

### Opioid analgesics

Prescription opioids have major drawbacks that limit their tolerability, effectiveness, and safety. Opioids produce disorienting psychoactive effects which can interfere with daily activities. Opioid use can cause constipation which produces significant discomfort. Repeated opioid use leads to adaptations in opioid receptor signaling, such as receptor desensitization, internalization, and augmented downstream signaling pathways, which are thought to differentially contribute to tolerance and limit effectiveness in treating pain (von Zastrow et al., 2003; Gintzler and Chakrabarti, 2006; Martini and Whistler, 2007). Activation of opioid receptors in circuits that control breathing induces strong respiratory depression that leads to death at high doses, with opioid-related deaths rising steadily over the past 20 years and continuing at epidemic levels (Rudd et al., 2016; Scholl et al., 2019). Coupled with the rewarding aspects of opioid signaling that reinforce drug consumption, respiratory depression is the most dangerous aspect of opioid analgesics, as it is responsible for the large number of opioid overdose deaths. There is thus an urgent demand for novel effective and tolerable treatment paradigms to lessen suffering of chronic pain patients, a mission that has been recently prioritized by the US National Institutes of Health (Collins et al., 2018).

### Endogenous opioids

Opioid receptors are expressed throughout the nervous system, including the cortex, midbrain, brainstem, spinal cord, and in the presynaptic terminals of the primary afferents of the dorsal root ganglion (le Merrer et al., 2009). Due to its prominence as the primary target of opioid analgesics, most studies of pain revolve around the μ-opioid receptor (MOR). However, the δ- and κ-opioid receptors (DORs and KORs) are also important in pain modulation (Fields, 2004; Corder et al., 2018). MORs are activated by the endogenous opioid neuropeptides enkephalin, beta-endorphin, and dynorphin. Enkephalins, of which there are two forms that differ in their C-terminal amino acid ([Met^5^]-enkephalin and [Leu^5^]-enkephalin), also activate DORs with similar affinity (Toll et al., 1998; Gomes et al., 2020). Beta-endorphin, which includes [Met^5^]-enkephalin at its N-terminus, is usually considered MOR-selective but can also activate DORs and KORs, with notable signaling bias toward downstream G-protein signaling compared to beta-arrestin signaling at MORs observed *in vitro* (Gomes et al., 2020). Several opioid peptides that can be described as short, C-terminally extended forms of [Met^5^]-enkephalin have also been isolated from mammalian brains; one of which (Met-enkephalin-Arg-Phe) has been recently demonstrated to act at MORs when released endogenously (Trieu et al., 2022). Several dynorphin peptides of different length and sequence are prominent in the mammalian nervous system. Although dynorphins are usually considered KOR agonists due their high affinity for KORs (especially the longer forms), they can also activate MORs and DORs at physiologically relevant concentrations (Toll et al., 1998; Gomes et al., 2020).

It is generally assumed that endogenous opioids produce pain relief through MOR activation. The most unequivocal experimental manipulation in humans implicating endogenous opioids in pain is the administration of naloxone, which is a non-specific opioid antagonist that acts on MORs, DORs, and KORs in a similar concentration range. Thus, endogenous opioids may impart some of their antinociceptive effects through activation of DORs and KORs, in addition to MORs.

### Pain processing circuits and their expression of opioid receptors

**FIGURE 1:**
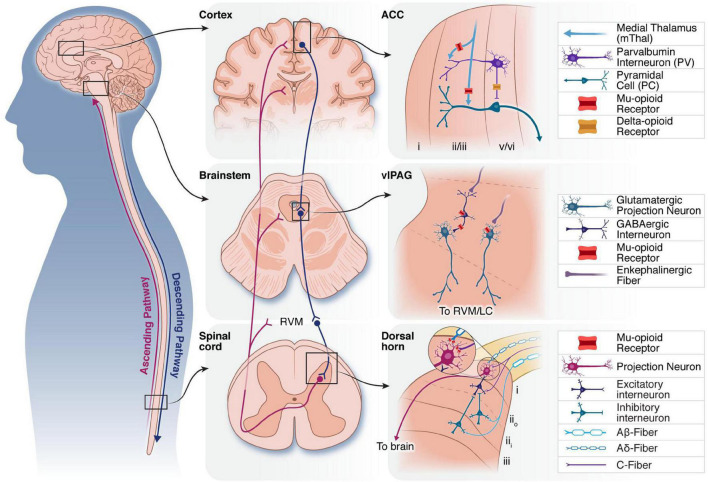
Overview of three neural structures that have been targeted by neurostimulation therapies. Schematic of ascending (purple) and descending (blue) pain modulatory pathways **(left)**. Middle: Macro level anatomy of the cortex, brainstem and spinal cord, showing key nodes in the ascending and descending pain modulatory pathways. Connections between the brainstem and spinal cord *via* the RVM are indicated. Right: Select synaptic connections and microcircuitry of the ACC, vlPAG and DH are shown. Mu-and delta-opioid receptors are expressed on cell bodies and pre-synaptic terminals of neurons throughout the pain neuraxis to modulate ascending and descending pain pathways. ACC, anterior cingulate cortex; RVM, rostroventromedial medulla; vlPAG, ventrolateral periaqueductal gray; LC, locus coeruleus; DH, dorsal horn.

Pain information is processed by two broad pathways: the ascending nociceptive pathway and the descending pain modulatory system (DPMS). The ascending pathway begins in peripheral nociceptors, which encode painful stimuli and synapse onto projection neurons and interneurons in the spinal cord dorsal horn (DH). Ascending pathways include the spinothalamic, spinomesencephalic, and spinoreticular tracts, which target the thalamus, midbrain areas such as the periaqueductal gray (PAG), and the brainstem reticular formation, respectively. Within the spinothalamic tract, subdivisions that target the lateral thalamus and onto the somatosensory cortices and insula are considered to mediate the sensory-discriminative aspects of pain (i.e., the sensory experience of pain involved in reflexive pain behaviors such as limb withdrawal in response to noxious stimuli). Spinothalamic subdivisions that target the medial thalamus and onto the anterior cingulate cortex (ACC) are thought to contribute to the affective percept of pain (i.e., the emotional-motivational experience of pain which is non-reflexive). The descending pain modulatory pathway begins in the PAG. Canonically, ventrolateral PAG (vlPAG) projects to the rostroventral medulla (RVM), which in turn sends projections to the DH to gate spinal outflow of incoming pain information. A brief overview of key brain areas that encode and modulate pain for the understanding of neurostimulation-induced analgesia follows. Schematics of the location, circuitry, and opioid receptor expression in brain areas within the descending and ascending pathways most relevant for current neurostimulation techniques for the treatment of chronic pain are shown in Figure 1.

#### Descending pathway

##### Periaqueductal gray

The PAG, a heterogenous midbrain region known for its roles in divergent behaviors such as defensive responses and vocalization (Behbehani, 1995), represents the first major hub in the DPMS. In the context of the pain, PAG receives and consolidates top-down input from numerous cortical and subcortical regions, including the prefrontal cortex (PFC), ACC, anterior insula, and amygdala (Hardy and Leichnetz, 1981; Bingel et al., 2006; Lu et al., 2016; Cheriyan and Sheets, 2018; Li and Sheets, 2018; Rozeske et al., 2018; Huang et al., 2019; Zhu et al., 2021). In addition to the RVM and nearby noradrenergic nuclei, the PAG displays broad ascending efferent projections to brain regions such as the thalamus, hypothalamus, and ventral tegmental area (Cameron et al., 1995a,b; Linnman et al., 2012; Ntamati et al., 2018). Though human tractography studies indicate some differences in PAG cortical connectivity between rodents and humans, midbrain and hindbrain connectivity is conserved, which is critical to our understanding of neurostimulation techniques that may harness descending pain modulatory mechanisms (Ezra et al., 2015; Menant et al., 2016).

In the rodent, the anatomy and function of the PAG opioid system has been extensively studied and recently reviewed by Bagley and Ingram, 2020. The canonical circuit by which opioids signal in the PAG follows a disinhibitory mechanism: MORs are highly expressed on local vlPAG GABAergic interneurons that provide tonic inhibition onto PAG projection neurons. In the presence of endogenous or exogenous opioids, these inhibitory inputs are suppressed by MOR signaling, leading to the disinhibition of glutamatergic PAG-RVM projections (Lau and Vaughan, 2014). The resultant activation of descending GABAergic, opioidergic and serotonergic RVM neurons directly inhibits spinal cord neurons to suppress nociception (Salas et al., 2016; Weiwei et al., 2021).

In line with this hypothesis, vlPAG microinfusion of glutamate receptor agonists and GABA receptor antagonists produces antinociception in rodents (Moreau and Fields, 1986; Jones and Gebhart, 1988; Jensen and Yaksh, 1989). More recently, modern chemogenetic methods in behaving rodents indicate that activation of glutamatergic vlPAG neurons or inhibition of GABAergic neurons is antinociceptive, while inhibition of glutamatergic neurons or activation of GABAergic neurons is pronociceptive, although the opioid dependence of this analgesia was not examined (Samineni et al., 2017). Local opioid infusion in the PAG, especially vlPAG, has long been noted for its strong antinociceptive properties in rodents (Yaksh, 1979; Jones and Gebhart, 1988; Jensen and Yaksh, 1989). MORs can also be found, however, in a subpopulation of PAG projection neurons (Wang and Wessendorf, 2002; Bagley and Ingram, 2020), suggesting that this accepted circuitry may not account for non-canonical or bidirectional signaling from PAG to RVM, which may involve competing facilitation and inhibition. Indeed, about half of RVM-projecting PAG neurons are actually hyperpolarized by MOR agonists (Osborne et al., 1996; Umana et al., 2017).

Using functional imaging in humans, PAG activity has been implicated in a multitude of functions, from pain-and placebo-related conditions to homeostatic bodily processes and the manifestation of negative emotional states in panic and depression (Zhao, 2008; George et al., 2019). For a comprehensive review of human functional imaging of PAG, we recommend the meta-analysis provided by Linnman et al., 2012. In brief, many studies have found pain-induced PAG activation in response to noxious stimuli such as heat, cold, pressure, and light touch on allodynic regions, as well as in chronic pain conditions such as neuropathic pain. PAG fMRI indicates its functional connectivity at rest with ACC and RVM (Kong et al., 2010), and this ACC-PAG interaction correlates with attentional analgesia and can be disrupted by opioid antagonists (Oliva et al., 2022). Placebo conditioning in humans increases PAG activity during the anticipation of a painful stimulus (Wager et al., 2004) and induces coupling of ACC and PAG activity that is sensitive to systemic naloxone (Eippert et al., 2009). Due to the abundance of opioid receptors expressed, PAG is thought to play a key role in pain modulation produced by exogenous and endogenous opioids. In humans, PET imaging of [11C]-carfentanil indicates a decrease in radiotracer binding and therefore an increase in PAG endogenous opioid signaling in response to pain (Zubieta et al., 2005) and placebo analgesia (Scott et al., 2008).

##### Rostroventral medulla

Rostroventral medulla (RVM) receives inputs from PAG and sends projections to the DH to modulate spinal signaling through GABAergic, serotonergic, and opioidergic mechanisms (Millan, 2002; François et al., 2017). RVM neurons are categorized as ON, OFF, and neutral cells based on their electrophysiological responses to noxious stimuli and during nocifensive responses. RVM receives input from the PAG and has recently been shown to receive synaptic connections from the parabrachial nucleus (Chen et al., 2017). RVM outputs relevant for pain modulation include the spinal cord and midbrain and brainstem noradrenergic nuclei (Clark and Proudfit, 1991a).

Like PAG, RVM is a known locus of exogenous and endogenous opioids in pain modulation (Bagley and Ingram, 2020). RVM neurons express opioid receptors in serotonergic and non-serotonergic neurons that project to the spinal cord (Gutstein et al., 1998; Wang and Wessendorf, 1999). Supporting a role for endogenous opioids, all three opioid receptor types are also expressed by terminals in the neuropil around RVM neurons (Kalyuzhny et al., 1996; Gutstein et al., 1998). RVM receives input from enkephalinergic terminals and some RVM neurons are enkephalinergic, including a subset of spinally-projecting GABAergic neurons (Khachaturian et al., 1983; Zhang et al., 2015). In addition to enkephalins, RVM receives dynorphinergic input from PAG and contains KOR-expressing spinally-projecting neurons that inhibit pain and itch *via* descending mechanisms (Nguyen et al., 2022). RVM may also contain dynorphin-expressing neuronal cell bodies (Menetrey and Basbaum, 1987). Application of opioids to the RVM leads to the increase in activity of antinociceptive OFF-cells and the decrease in spiking of pronociceptive ON-cells (Heinricher et al., 1994) as well as strong antinociception in rodents (Dickenson et al., 1979; Azami et al., 1982).

##### Noradrenergic cell groups

Rodent intrathecal pharmacological studies have long implicated spinal noradrenergic signaling as a key component in supraspinal influence on pain suppression (Yaksh, 1979; Proudfit and Hammond, 1981; Hammond and Yaksh, 1984; West et al., 1993). The locus coeruleus (LC) (A6), brainstem (A5), and midbrain (A7) noradrenergic cell groups display projections to the spinal cord in parallel with the RVM (Westlund et al., 1983, 1984; Clark and Proudfit, 1991b,c, 1993; Proudfit and Clark, 1991; Bruinstroop et al., 2012; Li et al., 2016; Hirschberg et al., 2017) and receive anatomical input from canonical DPMS nuclei PAG and RVM (Clark and Proudfit, 1991a; Bajic and Proudfit, 1999).

Locus coeruleus (LC) highly expresses opioid receptors (Pert et al., 1976) and LC neuron activity is directly suppressed by both endogenous and exogenous opioids (Williams et al., 1982). Opioid receptor expression in LC, A5, and A7 neurons appears to be limited to MORs (Williams and North, 1984; North et al., 1987; Guajardo et al., 2017), although a subset of presynaptic terminals in these areas have been shown to express DORs (Arvidsson et al., 1995; van Bockstaele et al., 1997; Holden et al., 1999; Erbs et al., 2015). Additionally, LC and the pericoerulear region are densely innervated by enkephalin-expressing terminals (Drolet et al., 1992). Microinfusion of morphine directly into the LC is antinociceptive in rodents (Bodnar et al., 1988).

##### Spinal cord

The spinal cord, especially the DH, is the ultimate target of the DPMS. Release of neuromodulators and neurotransmitters in the DH from descending sources modulates spinal outflow of ascending nociceptive information arriving from the periphery. Aδ and C nociceptive fibers terminate onto DH superficial laminae I projection neurons that respond to high threshold stimulation, as well as onto deeper layer V wide dynamic range projection neurons. Most neurons in the laminae II-III, however, are not supraspinally-projecting, but instead are excitatory or inhibitory interneurons that signal locally in the spinal cord. It is thought that descending fibers from the midbrain and brainstem can terminate onto primary afferent terminals, spinal interneurons, and spinal projection neurons to modulate the spinal circuit response to incoming pain information at multiple levels (Mannion and Woolf, 2000; D’Mello and Dickenson, 2008). In addition to neurotransmitters, spinal pain transmission is also modulated by a complicated combination of other neurochemicals such as neurokinins, CGRP, somatostatin, and opioids (Dickenson, 1995).

Endogenous opioid peptides and receptors play a substantial role in spinal cord pain-related activity. The rat spinal cord predominantly expresses MORs, but also exhibits some DORs and very low KOR expression. Within each of these receptor subtypes, all show predominant expression on presynaptic terminals entering the DH, with a smaller proportion on postsynaptic neurons (Besse et al., 1990; Dickenson, 1995). Recordings from DH neurons during intrathecal morphine application show that C and Aδ fibers that convey noxious information are the most highly inhibited by morphine, while the pain evoked activity of larger Aβ mechanosensory fibers is only mildly opioid-modulated (Dickenson and Sullivan, 1986; Heinke et al., 2011). Intrathecal application of enkephalin is analgesic (Yaksh et al., 1977), presumably due to activation of the same opioid receptors affected by morphine. Enkephalin- and dynorphin-immunoreactive cell bodies and fibers are present in the DH, suggesting that endogenous opioid peptides are released in the DH locally and by descending mechanisms (Seybold and Elde, 1980; Harlan et al., 1987; Marvizón et al., 2009; François et al., 2017). However, parsing the contribution of local and descending opioid release has been experimentally challenging.

#### Ascending pathway

##### Thalamus

The thalamus receives nociceptive information directly from the spinal cord and relays it to the cortex (Ab Aziz and Ahmad, 2006). The spinothalamic tract conveys information about non-noxious and noxious stimuli to the lateral and medial thalamus. The lateral thalamic ventral posterolateral (VPL) and ventral posteromedial (VPM) nuclei project to the somatosensory cortex and relay tactile, proprioceptive, and nociceptive signals from the body and face, respectively (Monconduit et al., 1999; Alitto and Usrey, 2003). Medial thalamic nuclei receive additional nociceptive information from ascending spinal tracts. These nuclei transmit information thought to be related to the affective components of pain to areas involved in emotional processing, such as the ACC and the insular cortices (Friedman and Murray, 1986). A study in rats found a functional correlation between medial thalamus and ACC activity during electrical stimulation, supporting the idea that thalamus conveys information on the affective components of pain through this projection (Shyu et al., 2004). Among the medial thalamic nuclei, the mediodorsal nucleus (MD) is the major source of inputs to the ACC. Also implicated in pain processing is the medial thalamic nucleus submedius (Sm), which projects to the ventrolateral orbital cortex (VLO) and on to the PAG, a pathway that has been shown to mediate antinociception (Zhang et al., 1995; Huang et al., 2021). Imaging and electrophysiology studies in both animals and humans have also found that, like ACC, the MD is hyperactive in chronic pain conditions (Whitt et al., 2013; Meda et al., 2019). In mice with neuropathic pain, optogenetic activation of MD inputs to ACC induces behavioral avoidance and is considered aversive (Meda et al., 2019).

A meta-analysis of published fMRI data in humans with acute, experimentally-induced and chronic pain showed that the thalamus is active in both conditions (Friebel et al., 2011). Chronic pain patients show altered thalamic regional cerebral blood flow (rCBF) and several imaging studies suggest that altered thalamic activity is involved in the development of neuropathic pain (Witting et al., 2001; Casey et al., 2003). Studies in animal models of neuropathic pain have also shown a correlation between chronic pain and changes in biochemistry and immediate early gene expression in the thalamus (Narita, 2003).

Opioid receptors are widely expressed in the thalamus. High levels of MOR mRNA are observed in several thalamic nuclei, including the medial habenula, laterodorsal, paraventricular, centromedial, and reuniens nuclei. DOR mRNA expression is also observed in the thalamus, but KOR mRNA expression is limited to fewer nuclei in the paraventricular and zona incerta (Mansour et al., 1994; Erbs et al., 2015). In rodent brain slices, thalamic output to ACC and dorsal striatum is suppressed in the presence of a MOR agonist, indicating the sensitivity of thalamic output to opioids and suggesting the attenuation of noxious information relay to cortex during opioid treatment (Birdsong et al., 2019). In rodents, pharmacological blockade of MORs in the dorsal midline thalamus induced a fear memory extinction deficit (Bengoetxea et al., 2020), while stimulation of MORs caused increased locomotor activity associated with decreased freezing extinction. These data suggest that targeting dorsal midline thalamus MORs could have therapeutic effects on stress-related and anxiety disorders. Animal research using both electrophysiology and EEG points to the medial thalamus as the primary site of morphine action (Linseman and Grupp, 1980). Indeed, morphine microinfused in the medial or intralaminar thalamic nuclei has been shown in a small number of rodent studies to produce analgesia (Carr and Bak, 1988; Wang et al., 2006; Erfanparast et al., 2015). Consistently, studies in both humans measuring [11C]diprenorphine binding *via* PET imaging and rodents have found lower opioid receptor availability in chronic pain conditions in the thalamus, ACC, posterior temporal and orbitofrontal cortices, as well as in the posterior midbrain (Thompson et al., 2018).

##### Anterior cingulate cortex

The ACC refers to a subregion of frontal cortex with heterogenous subdivisions that are differentially involved in the affective, cognitive, and emotional components of pain processing (Bush et al., 2000; Vogt, 2005; Heilbronner and Hayden, 2016). In humans, ACC receives inputs from the anterior insular cortex (aI) (Peltz et al., 2011; Wiech et al., 2014) and amygdala (Sharma et al., 2020). It receives ascending noxious sensory information mainly *via* the medial thalamic nuclei (Xiao and Zhang, 2018). The ACC pain-aversive response can be increased by inputs from the primary somatosensory cortex on a subset of ACC neurons (Singh et al., 2020). Several pieces of evidence suggest that projections from ACC to the brainstem, specifically through the PAG or by way of the medial thalamic nuclei, are important for the cortical contribution to opioid analgesia and to placebo analgesia (Hardy and Leichnetz, 1981; Royce, 1983; Devinsky et al., 1995). ACC also sends reciprocal projections to the amygdala (Allsop et al., 2018) and insular cortex; while functional connectivity between these regions is associated with negative affective states (Shao et al., 2018), the role of this circuitry in the emotional and affective components of pain remains to be determined.

Early single neuron recordings in cingulotomy patients showed that ACC neurons respond selectively to mechanical and thermal painful stimuli, but not to innocuous stimuli (Hutchison et al., 1999). Likewise, single-unit recordings in rabbits demonstrate that ACC neurons which respond to noxious stimuli have diffuse receptive fields covering the entire body (Sikes and Vogt, 1992). In non-human primates, ACC neurons were reported to encode the integration of nociception, specifically the anticipation of pain following cutaneous electric stimulation (Koyama et al., 1998). Interestingly, ACC activation has also been observed during placebo-induced analgesia (Wager et al., 2004), though this activation may occur in a different substructure than that activated by noxious stimuli. Subsequent human fMRI and PET studies further confirm that ACC is activated by noxious stimuli (Kwan et al., 2000) and the response magnitude correlates with stimulus intensity and changes in the perceived unpleasantness of painful stimuli (Vogt et al., 1996; Rainville et al., 1997; Tölle et al., 1999). Together, these findings confirm that nociceptive stimuli activate ACC across species.

Arguing against a simple role for the ACC in nociception, patients with ACC lesions experience reduced pain-related unpleasantness and reduced avoidance of noxious stimuli, but their ability to identify intensity and location of noxious stimuli remains intact (Foltz and White, 1962; Ballantine et al., 1967; Wayne Hurt et al., 1974). Similarly, microinjection of excitatory amino acids into the ACC in naïve rodents elicits conditioned place aversion without altering pain thresholds (Johansen and Fields, 2004), while ACC lesions eliminate the aversiveness of neuropathic pain but not stimulus-evoked hypersensitivity (Qu et al., 2011). These findings argue against the role of ACC in nociceptive processing *per se*. Instead, several studies in both humans and rodents have shown that ACC contributes to the unpleasantness of pain (Seminowicz et al., 2009; Fuchs et al., 2014; Bliss et al., 2016). Functional and structural alterations of ACC, such as hyperactivation and reduction of gray matter, have been observed in neuropathic patients and are associated with emotional and psychological pain (Rodriguez-Raecke et al., 2009; Bushnell et al., 2013).

Early human studies reported high [3H]diprenorphine binding in the ACC of healthy subjects but a reduction in patients with central post-stroke pain (Willoch et al., 1999), suggesting that opioids can directly impact aspects of pain processing by binding ACC opioid receptors (Vogt et al., 1995; Jones et al., 1999). Further receptor-imaging studies confirm the involvement of ACC in opioid-dependent analgesia and, intriguingly, suggest a role in placebo analgesia (Petrovic et al., 2002). PET studies performed with [11C]Carfentanil observed endogenous ACC opioid release during placebo analgesia and the consequent endogenous opioid-induced ACC activation correlated with a reduction in pain affect during a sustained painful stimulus (Zubieta et al., 2001). Consistent with this, rodent ACC morphine microinjection selectively suppresses pain affect but not withdrawal responses (LaGraize et al., 2006; Gomtsian et al., 2018).

Opioid receptors are abundantly expressed in the ACC, with MOR expression most prominent in superficial layers (Vogt et al., 1995). MORs are expressed by both cortical neurons and afferent axons from subcortical regions. Presynaptic MORs are predominant on thalamic axonal projections to the ACC (Vogt et al., 1995). This distribution pattern led to the idea that endogenous opioids can regulate nociception by inhibiting the thalamocortical afferents in the ACC or by modulating the activity of interneurons and projection neurons (Navratilova et al., 2015). This model has been recently expanded upon by examining the thalamo-cortico-striatal circuit (Birdsong et al., 2019), whose involvement in pain processing was first described by Rainville et al. (1997). Thalamic inputs to ACC are potently inhibited by MOR agonists, but ACC inputs to dorsomedial striatal neurons are not affected. In contrast, DOR agonists disinhibit ACC pyramidal neurons and allow for the excitation of ACC inputs onto striatal medium spiny neurons. These mechanisms are mediated by different receptors and suggest that opioid-mediated attenuation of nociceptive information transfer to ACC from thalamus may be a primary mechanism by which opioids reduce the negative affective component of pain.

##### Prefrontal cortex

While most frequently studied in the context of executive cognitive function, recent evidence has begun to implicate the PFC in processing acute nociceptive stimuli and in the development of chronic pain. Within the PFC, the dorsolateral PFC (dlPFC) is considered a master regulator of higher order cognitive functions and is also involved in the cognitive and affective modulation of pain (Lorenz et al., 2003), including placebo analgesia (Petrovic et al., 2002). Functional imaging in humans with acute and chronic pain reveal that PFC activity correlates with the activity of pain-implicated regions above, including ACC, insula, and thalamus (Apkarian et al., 2005). Further, it has been posited that PFC-PAG output and reciprocal PFC connections with the amygdala play a role in antinociception, whereas thalamocortical PFC input and PFC output to the basal ganglia may contribute to pain chronicity (Ong et al., 2019). Previous fMRI studies have found that the magnitude of placebo-induced dlPFC activity correlates with an increase in PAG activity, supporting the idea that this circuit is involved in expectancy-based placebo (Wager et al., 2004, 2007). The prelimbic cortex in rodents is often included in definitions of the rodent PFC, and while not considered homologous to dlPFC in primates (Laubach et al., 2018), recent work has revealed a role for this structure in pain processing. Specifically, inflammatory pain decreases both basal firing rate and evoked nociceptive responses in prelimbic neurons (Dale et al., 2018), while inhibition of prelimbic neurons and their outputs to the nucleus accumbens enhances pain responses (Zhou et al., 2018).

The effects of opioids in the PFC are less well-characterized. Rodent PFC neuronal activity has been shown to be opioid sensitive (Williams and Zieglgänsberger, 1981; Giacchino and Henriksen, 1998), while in humans, PET imaging implicates PFC endogenous opioid signaling in placebo-induced analgesia (Wager et al., 2007). Caution is required, however, when attempting to draw parallels between the rodent and human PFC as expansion over the course of evolution has led to more distinct functions and subregions within the human PFC as compared to the rodent (Carlén, 2017; Laubach et al., 2018), with rodents lacking a specific homologue of the dlPFC. Nonetheless, important findings for the implications for PFC in pain signaling may still be gleaned by carefully designing and interpreting experiments and corroborating findings across experimental models.

## Neurostimulation therapies for chronic pain

It is now well-established that the widespread adoption of prescription opioids for the treatment of chronic pain has been instrumental in driving the ongoing opioid epidemic. The continuing burden of untreated chronic pain on patients underscores the need for safe and effective pain therapies. Neurostimulation therapies that target peripheral or central pain mechanisms are promising alternatives for managing medically refractory pain. However, these therapies are hampered by inconsistent pain relief across patients and frequently diminishing analgesic effects over time. Across all neurostimulation therapies, we do not currently understand the physiological mechanisms of action by which these therapies provide pain relief. A clear understanding of the mechanisms of stimulation-induced analgesia is crucial to improve the efficacy of these therapies.

### Overview of neurostimulation for chronic pain

**FIGURE 2:**
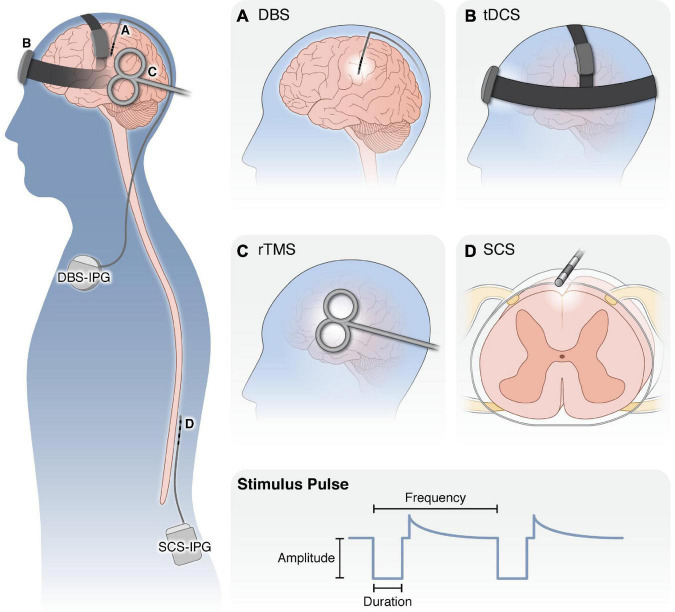
Overview of neurostimulation modalities for the treatment of chronic pain. (Left) Schematic of application of neurostimulation devices for the treatment of chronic pain. **(A)** DBS electrodes are surgically targeted to specific brain nuclei (i.e., ACC, midline thalamus, PAG) with an external pulse generator. Following optimization of stimulation settings, the pulse generator and leads are internalized under the clavicle to deliver electrical stimulation to the brain. **(B)** With tDCS, small amounts of electric current are applied externally *via* electrodes held in place against the scalp. **(C)** rTMS is applied with an external electromagnetic coil to generate an electromagnetic field in the underlying cortical regions. Both tDCS and rTMS are applied for 20–60 min over repeated sessions without requiring anesthesia. **(D)** SCS employs implanted electrodes in the epidural space to apply electrical current to the spinal cord. Similar to DBS, SCS patients undergo a trial period to ensure adequate pain relief before the pulse generator and leads are internalized in the posterior flank. (Bottom) For all modalities, several properties of the stimulus waveform can be modulated, including the waveform shape, pulse amplitude, duration, and frequency, as well as whether it is applied continuously, in regular burst patterns or in a closed-loop manner in response to neural activity or patient control. DBS, deep brain stimulation; tDCS, transcranial direct current stimulation; rTMS, repeated transcranial magnetic stimulation; SCS, spinal cord stimulation; IPG, implanted pulse generator.

Neurostimulation therapies (Figure 2) are non-addictive, reversible strategies for managing intractable chronic pain. Neurostimulation therapies aim to modulate neural activity through targeted delivery of electrical stimuli to specific regions of the nervous system. In the clinical context, the term “neuromodulation” commonly refers to electrical neurostimulation therapies, but may also refer to targeted drug delivery (e.g., intrathecal pumps), radiofrequency ablation therapies, or modulation of neural activity *via* ultrasound, which are outside the scope of this review. We use the terms “neuromodulation” and “neurostimulation” interchangeably to describe therapies which use electrical stimulation of the nervous system to treat neurological disorders.

Neurostimulation therapies range in invasiveness. Non-invasive therapies, such as transcranial direct current stimulation (tDCS), place electrodes on the scalp or magnetic coils proximal to the head. Invasive neurostimulation therapies, such as deep brain stimulation (DBS) or spinal cord stimulation (SCS), involve placing small electrode arrays in the body near the neural structure of interest, which are connected to implantable pulse generators. After electrode placement, a clinician programs the stimulus pulse (i.e., sets the stimulus pulse amplitude, duration, and frequency) to maximize therapeutic effect while minimizing unwanted side effects. These stimulation parameters may be adjusted at follow-up visits to ensure consistent therapeutic benefit.

Neurostimulation has emerged in the past 60 years as an effective therapeutic approach to treating pain and other disorders (Bittar et al., 2005; Moisset et al., 2020). Based on the premise that pain percept is encoded by aberrant patterns of neural activity, the objective of neurostimulation is to alter neural activity in a way that minimizes the experience of pain. Melzack and Wall’s Gate Control Theory of Pain formed the scientific basis for the first modern uses of electrical stimulation-induced pain relief in humans (Melzack and Wall, 1965). This theory suggests that driving the activity of large-diameter afferents may produce pain relief by increasing the activity of inhibitory interneurons in the spinal cord DH. Only 2 years after the publication of the Gate Control Theory, Wall and Sweet demonstrated analgesia *via* peripheral nerve stimulation (Wall and Sweet, 1967), and Shealy and colleagues demonstrated analgesia *via* electrical stimulation of the dorsal columns of the spinal cord (Shealy et al., 1967). Conventional neurostimulation theory suggests that extracellular electrical stimulation induces action potentials (APs) in myelinated axons at lower stimulus amplitudes than other neural structures (e.g., non-myelinated axons, cell bodies) (Rattay, 1986, 1999; McIntyre and Grill, 1999). Therefore, electrical stimulation of peripheral nerves and the dorsal columns likely provides analgesia by driving the activity of myelinated tactile afferent axons and feed-forward pain-gating circuitry (Mendell, 2013; Braz et al., 2014; Duan et al., 2018).

The past several decades have produced many innovations in stimulation-induced analgesia. Therapies such as spinal cord stimulation (SCS) are most commonly indicated for neuropathic limb pain conditions, such as failed back surgery syndrome and complex regional pain syndrome. Modern neurostimulation approaches have also been investigated to treat central chronic pain syndromes, such as post-stroke and phantom limb pain (Bittar et al., 2005; Moisset et al., 2020). Furthermore, novel stimulation targets (e.g., deep brain stimulation (DBS) of the ACC (Spooner et al., 2007)) and stimulus pulse paradigms [e.g., burst SCS (de Ridder et al., 2013)] are hypothesized to modulate the neural activity associated with the affective component of pain, rather than affecting circuits associated with the sensory component (e.g., the spinal cord DH). Recent years have seen numerous promising innovations in neurostimulation for pain, and these modalities of exogenous electrical stimulation likely have broad effects across the pain neuraxis, which are not limited to circuits being directly stimulated. This property poses additional challenges to understanding the specific therapeutic mechanisms underlying each neurostimulation technique. Therefore, understanding how different neurostimulation therapies affect specific circuits, such as opioidergic circuits, is crucial to understanding the mechanisms that will ultimately be necessary for optimizing the design and implementation of each therapy.

### Spinal cord stimulation

Spinal cord stimulation (SCS) is the most common neurostimulation therapy, with more than 50,000 SCS systems implanted each year (Sdrulla et al., 2018). SCS is primarily indicated for chronic neuropathic pain of the trunk or limbs which is refractory to conventional medical management (Kumar et al., 2007). SCS is achieved by implanting an electrode array in the dorsal epidural space, either *via* percutaneous implantation of a cylindrical electrode array, or by implanting a paddle electrode array which requires a laminectomy (Sears et al., 2011). Traditionally, SCS is applied with stimulus pulse frequencies between 40 and 60 Hz, pulse durations between 200 and 600 μs, and pulse amplitudes on the order of several Volts or milliamps for voltage-and current-controlled stimulation, respectively (Kumar R. et al., 1998; Kapural et al., 2015; Malinowski et al., 2020). Recent innovations in SCS technology apply novel stimulus pulse paradigms, particularly with regards to stimulus pulse frequency (Lempka and Patil, 2018). However, few studies have provided evidence regarding the involvement of endogenous opioid mechanisms in analgesia achieved with these novel SCS therapies. Therefore, we will focus our discussion on the possible opioidergic mechanisms of conventional SCS. Furthermore, to the extent that peripherally-targeted neurostimulation therapies such as peripheral nerve stimulation (PNS) (Helm et al., 2021) and dorsal root ganglion stimulation (DRGS) (Deer et al., 2017) engage the CNS, they are hypothesized to directly stimulate similar neural targets as conventional SCS (Lin et al., 2020; Graham et al., 2022). Accordingly, in addition to potentially modulating action potential propagation in nociceptors, these therapies likely engage similar central analgesic mechanisms as with conventional SCS.

Conventional SCS applied with pulse frequencies between ∼40 and 60 Hz evokes paresthesia (i.e., tingling or pins-and-needles sensations) in the area of the body targeted by stimulation. The goal of stimulator programming is to overlap these evoked paresthesias with the patient’s painful region (North et al., 1991). Conventional SCS induces bidirectionally propagating action potentials (APs) in Aβ axons in the dorsal columns (Struijk et al., 1991; Holsheimer, 2002; Zhang et al., 2014; Lempka et al., 2020; Rogers et al., 2022). Antidromically propagating APs enter the dorsal horn caudal to the spinal level where SCS is applied, where they likely provide pain relief by activating feed-forward pain-gating circuitry in the spinal cord. Orthodromically propagating APs are likely responsible for SCS-induced paresthesia (Moffitt et al., 2009) and enter the brain at the brainstem dorsal column nuclei. It is possible that SCS simultaneously engages the endogenous opioid system both *via* orthodromically propagating APs to the brain and antidromically propagating APs into the DH.

Several brain structures related to the endogenous opioid system have been implicated in the supraspinal mechanisms of action of SCS, such as the PAG, RVM, and thalamic VPL nucleus (Sivanesan et al., 2019). Many studies have examined the role of the DPMS, particularly the GABAergic and serotonergic components, in SCS-induced analgesia (Cui et al., 1996; Song et al., 2009, 2011). Early work in four patients suggested that SCS-induced analgesia is not reversed by naloxone administration, suggesting opioid-independent mechanisms (Freeman et al., 1983). However, this study examined a limited number of patients, and subsequent preclinical work has demonstrated RVM activation during SCS, a structure known to be crucial in endogenous opioid release (Dejongste et al., 1998), leaving the role of opioidergic circuits in SCS-induced analgesia unclear.

In more recent preclinical work, SCS applied to the cervical spinal cord caused dynorphin release in spinal segments caudal to the stimulation site (Ding et al., 2008), suggesting a potential role for segmental opioid release in SCS. In addition, SCS-induced analgesia in rats can be abolished by systemic naloxone, with both SCS-frequency and naloxone-dose dependent effects (Sato et al., 2013). A naloxone dose of 3 mg/kg/h reversed the effects of 4 Hz SCS, but the dose had to be increased to 10 mg/kg/h to reverse the analgesic effects of 60 Hz SCS. Interestingly, administering the DOR antagonist naltrindole abolished analgesia induced by 60 Hz but not by 4 Hz SCS. Finally, a recent preclinical study simultaneously applied SCS and the cholecystokinin (CCK) receptor antagonist proglumide (Inoue et al., 2017). While CCK receptor antagonists typically enhance opioid-dependent analgesia, co-application of SCS and proglumide did not provide enhanced analgesia compared to a single therapy alone. Taken together, these data present a murky picture regarding opioid-dependent analgesia during SCS, warranting continued study into both the involvement of endogenous opioids in SCS-induced analgesia and how SCS pulse parameters influence the engagement of these mechanisms.

### Deep brain stimulation

Deep brain stimulation (DBS) is a surgical therapy whereby electrode arrays are implanted in discrete nuclei in the brain. Current is then passed through these electrode contacts through a fully implanted pulse generator to manipulate brain activity. Due to its invasiveness, DBS is typically reserved as a late-stage intervention after pharmacological and behavioral treatments have proven ineffective. Brain regions targeted for DBS are often historically identified as sites at which surgical lesions provide some relief for a disorder. Relative to ablative surgery, DBS is reversible and individually programmable, enabling stimulation parameters to be titrated for each patient. Although most commonly used for treatment of movement disorders, indications for DBS have recently expanded to include major depressive disorder, obsessive compulsive disorder, Tourette syndrome, cluster headache, and chronic pain. We focus our discussion on three brain sites that have been targeted clinically for pain relief and highlight evidence for involvement of opioidergic mechanisms in the therapeutic effects of DBS applied to these brain targets.

#### Periaqueductal gray-deep brain stimulation

When targeting PAG, DBS electrodes are placed bilaterally or contralaterally to the site of pain. Some studies indicate that even unilateral electrode placement provides a largely generalized pain relief described as a feeling of warmth and analgesia (Hosobuchi et al., 1977; Boccard et al., 2015). Across multiple case studies, PAG-DBS has proven effective in patients with “nociceptive pain” (Kumar and Wyant, 1985; Levy et al., 1987; Gybels and Kupers, 1990; Kumar et al., 1990), referring to pain generated through ascending dorsal horn input, such as peripheral neuropathic pain, spinal cord injury, plexopathy or phantom limb pain (Prévinaire et al., 2009; Subedi and Grossberg, 2011). Conversely, PAG-DBS exhibits much lower efficacy in centrally generated pain (e.g., post-stroke pain or headache) (Levy et al., 1987; Kumar et al., 1990; Gray et al., 2014; Kashanian et al., 2020). PAG-DBS was largely abandoned in 2000 after two large scale clinical trials (206 total patients) failed to meet clinical endpoints (Coffey, 2001). However, several design and interpretation issues have been raised concerning these studies, including the absence of randomization or placebo control, heterogeneity of the initial pain condition, and attrition of patients from the study which reduced statistical power to detect treatment differences (Shirvalkar et al., 2020). Critically, most data on PAG-DBS has been collected in case series or small clinical trials, without proper randomization or double blinding, the latter of which is arguably unfeasible due to PAG-DBS-induced paresthesia. Though its popularity has decreased, PAG-DBS is still used clinically to treat patients who are treatment refractory with good overall outcomes (Boccard et al., 2013). In the future, patient selection will be a key focus point for refinement to optimize treatment efficacy (Farrell et al., 2018; Frizon et al., 2020).

The therapeutic effects of PAG-DBS are frequency-dependent, with frequencies between 5 and 25 Hz being more efficacious than frequencies above 50 Hz (Nandi et al., 2002; Hentall et al., 2016). Interestingly, patients tended to prefer stimulation frequencies as low as 0.67 Hz (Jermakowicz et al., 2017) and between 5 and 35 Hz (Nandi and Aziz, 2004) when given the opportunity to blindly tune the parameters of their own DBS. It is interesting to note that pain-relieving stimulation in the 5 to 25 Hz range is within the physiological firing frequency of PAG neurons (Yu et al., 2021) and stands in sharp contrast to frequencies classically used to treat movement disorders, which are typically above 100 Hz (Creed, 2018). This supports the interpretation that intermittent activation of PAG descending projections with DBS applied at a physiological firing rate could induce its effects through downstream opioid release.

In rodents with nerve injury, electrical stimulation of vlPAG was effective in reducing spontaneous pain behaviors and mechanical allodynia even 30–40 min after stimulation (Lee et al., 2012). A similar study using acute noxious stimuli found that unilateral vlPAG stimulation produces significant bilateral analgesia in rodents (Wang N. et al., 2016). Both studies state that the mechanism of this analgesia is still unclear, although opioids have been identified as a probable factor due to the concentration of MORs in PAG (Wang and Wessendorf, 2002; Loyd et al., 2008) and the finding that naloxone reverses some of the PAG stimulation-induced analgesia (Mayer et al., 1971; Akil et al., 1976; Morgan et al., 1991). Further downstream, the role of endogenous opioid release in the RVM for antinociception achieved by pharmacological and electrical activation of PAG has been assayed in preclinical models. PAG microinjection of GABA receptor antagonists (to cause PAG disinhibition), morphine, and non-opioid painkillers leads to antinociception that can be blocked by RVM microinfusion of naloxone (Llewelyn et al., 1984; Aimone and Gebhart, 1986; Kiefel et al., 1993; Roychowdhury and Fields, 1996; Vasquez and Vanegas, 2000). The role of endogenous opioid activity in the spinal cord with activation of DPMS by PAG and RVM electrical stimulation is still unclear; these stimulation interventions produce antinociception that can be blocked by intrathecal naloxone in some studies, while others have found a lack of an effect on antinociception by spinal opioid antagonism (Aimone et al., 1987; Miller and Proudfit, 1990; Morgan et al., 1991).

Clinical studies also suggest a role of endogenous opioids in PAG-DBS-induced analgesia. Early studies found that treatment with systemic naloxone blocks the analgesic effects of PAG-DBS in humans (Adams, 1976; Hosobuchi et al., 1977). A more recent study investigating dlPAG DBS-produced local field potentials also found that naloxone reversed the analgesia while increasing the 30–60 Hz band power measured at the same site, but this experiment was restricted to only two human subjects (Pereira et al., 2013). However, in a study of 45 patients with electrodes implanted in the PAG or periventricular gray (PVG), the attenuation of PAG-DBS pain relief by naloxone was similar in magnitude in both active and sham DBS conditions, suggesting the effect of naloxone may not specifically block PAG-DBS, but may instead enhance subjective pain ratings independent of stimulation (Young and Chambi, 1987). A study utilizing PET imaging to observe PAG opioid release found an increase in endogenous release during DBS, but it was not correlated with subjective analgesia (Sims-Williams et al., 2017). Furthermore, upon naloxone treatment, analgesia was still observed, with no significant effect to ongoing pain scores.

Additionally, it has been reported that patients may develop tolerance to chronic PAG-DBS stimulation and cross tolerance to opioids such that morphine becomes less effective after chronic PAG stimulation, suggesting occlusion of descending pain modulatory pathways and endogenous opioid release (Hosobuchi, 1986). However, other studies of PAG-DBS in humans have found tolerance to stimulation in other brain regions that are not presumed to function through endogenous opioid signaling and a lack of cross tolerance to morphine in chronic PAG-DBS (Young et al., 1985; Young and Chambi, 1987; Duncan et al., 1991). Finally, initial reports of endogenous opioid release driven by PAG stimulation in humans found increased enkephalin and beta-endorphin in cerebrospinal fluid of patients that had a positive, pain-relieving response to stimulation (Akil et al., 1978; Hosobuchi et al., 1979). Follow-up studies, however, found that this effect may be due to artifacts in immunoreactivity assays caused by contrast media (Dionne et al., 1984; Fessler et al., 1984). As a result of these collective studies, involvement of endogenous opioid peptides in PAG-DBS-driven analgesia remains unresolved.

#### Thalamus-deep brain stimulation

Compared to PAG-DBS, DBS in the sensory thalamus is thought to be more effective for deafferentation pain (Bittar et al., 2005), which is caused by damage to the peripheral or central nervous system that causes the loss of normal incoming pain signals. Examples of this type of pain include post-stroke pain, spinal cord injury, and facial anesthesia dolorosa (Hosobuchi et al., 1973; Adams et al., 1974). The theory behind the effectiveness of sensory thalamus DBS for this type of pain is that deafferentation pain is caused by a lack of normal proprioceptive information reaching the thalamus, which is combated by direct stimulation of VPL and VPM (Duncan et al., 1991). Additionally, stimulation may modulate the altered firing patterns in the sensory thalamus that are found in chronic pain patients (Dostrovsky, 2000; Moisset et al., 2020). When targeting sensory thalamus, stimulating electrodes are typically placed contralaterally and somatotopically according to the location of the painful area, and stimulation produces paresthesia in that area that masks pain (Hosobuchi et al., 1973; Boccard et al., 2015; Moisset et al., 2020). Comparatively, studies of sensory thalamic-DBS often use higher stimulus pulse frequencies than PAG-DBS, with frequencies falling between 50 and 100 Hz (Bittar et al., 2005; Moisset et al., 2020).

Deep brain stimulation (DBS) of medial thalamic centromedian-parafascicular nuclear complex (CM-Pf) has been attempted in humans under the assumption that this stimulation may activate descending pain modulatory opioidergic or non-opioidergic mechanisms, as well as drive a sensory feedforward loop with cortical targets (Andy, 1980; Duncan et al., 1991). While this manipulation appeared to be effective in a small cohort of patients with painful dyskinesia (Andy, 1980), other studies have produced variable results on reported painfulness and report a variety of potentially unpleasant side effects (Thoden et al., 1979; Hollingworth et al., 2017). Interestingly, a recent case study in 3 patients refractory to conventional neuromodulatory therapies found potential therapeutic benefits of dual stimulation of CM-Pf and PAG/PVG using a single electrode at different frequencies (Hollingworth et al., 2017).

The different electrical stimulation parameters of successful PAG-and thalamic-DBS strongly suggest that these two therapies exert their effects through distinct neural mechanisms. Early neurostimulation trials provide further evidence for this distinction. Specifically, responsiveness to morphine is used throughout the literature to select patients for PAG-or thalamic-DBS. Patients that respond moderately well to morphine are selected for PAG-DBS, while those that do not respond well to high doses of morphine are still able to find pain relief *via* thalamic-DBS whereas PAG-DBS would be ineffective (Hosobuchi, 1986). Along these lines, centrally generated pain is attenuated by thalamic-DBS, whereas PAG-DBS is not effective. These findings, coupled with the observation of low-threshold spontaneous discharge patterns in midline thalamic nuclei associated with pain states (Andy, 1983), lead to the hypothesis that thalamic-DBS produces a “functional lesion” by inducing depolarization block and inactivating low threshold discharging neurons surrounding the stimulation electrode. This “functional lesion” mechanism has also been proposed to account for the anti-dyskinetic effects of subthalamic nucleus-DBS applied for Parkinson’s disease, which shows pathological burst activity that correlates with onset of motor symptoms (Lobb, 2014). If an analogous mechanism of thalamic-DBS were confirmed, it presents the opportunity to trigger thalamic-DBS in response to nociceptive-related spontaneous discharge patterns of thalamic nuclei. Such closed-loop stimulation protocols have been increasingly adopted with STN-DBS for Parkinson’s disease and have the advantage of reduced off-target effects and extended battery life by requiring only intermittent stimulation.

#### Anterior cingulate cortex-deep brain stimulation

In contrast to PAG and thalamus which have been targeted with electrical stimulation for pain relief for over 30 years, DBS of dorsal ACC (dACC) has only recently emerged as treatment for neuropathic pain. In an initial case report (Spooner et al., 2007), a single patient with neuropathic pain resulting from a spinal cord injury received bilateral dACC-DBS electrodes and a unilateral electrode in the PVG. In this patient, DBS applied to the dACC at 130 Hz provided superior pain relief, mood improvement, and reduction in medication usage compared to PVG-DBS applied at 20 Hz. This treatment resulted in reduced pain as assessed *via* visual analog scale (VAS) pain ratings and pain medication usage. This patient also showed improved mood in terms of reduction of fear, anxiety, and depression, suggesting that dACC stimulation works at least in part by targeting pain affect.

Anterior cingulate cortex (ACC) stimulation in rodents can produce diverse behavioral effects depending on stimulus pulse frequency and which neuronal subtypes are stimulated. Unilateral electrical stimulation of the rodent ACC with intermittent trains of 100 Hz pulses (200 ms inter-train interval) induced fear-like freezing responses (Tang et al., 2005). Optogenetically activating ACC Thy1 + neurons at 20 Hz induced anxiodepressive behaviors, but did not increase the hindpaw withdraw threshold to mechanical stimuli (Barthas et al., 2015). Optogenetic activation at 10 Hz of CaMKII + excitatory ACC neurons (which partially overlap with the Thy1 + population) increased paw withdrawal thresholds in naïve mice, while inhibition reversed inflammatory pain-induced behavior (Kang et al., 2015). Further, nociceptive responses have been demonstrated to be attenuated in rodents following optogenetic and chemogenetic activation of subsets of ACC interneurons (Gu et al., 2015; Kang et al., 2015; Shao et al., 2021). These findings suggest that heterogeneity in both function, topography, and cellular architecture contribute to the diverse behavioral responses produced by ACC stimulation.

Clinical applications of ACC-DBS are typically applied at stimulation frequencies of approximately 130 Hz and stimulus pulse widths around 450 μs (Boccard et al., 2014, 2017). The efficacy of ACC-DBS has been shown for patients suffering from failed back surgery syndrome, poststroke pain, brachial plexus injury, cervical spinal cord injury, head injury, and pain of unknown origin (Boccard et al., 2014). Interestingly, some patients receiving ACC-DBS do not report significant reductions in pain as measured by numerical rating scales. However, many ACC-DBS patients report improvements in metrics related to the affective component of pain as well as overall improvements in quality of life and describe their pain as being “separate from them” or “not distressing” (Boccard et al., 2017).

Due to its novelty, there are few published studies on ACC-DBS mechanisms of action. However, the ACC projects to many pain matrix structures, such as amygdala and PAG (Shi et al., 2022). Therefore, it is possible that the analgesic effects of ACC-DBS are due to postsynaptic DPMS engagement. MORs are present both on local ACC cells and afferents (particularly from the thalamus) terminating in the ACC (Vogt et al., 1995). Furthermore, it is understood that terminating afferents are highly excitable near DBS electrodes (Bower and McIntyre, 2020). This suggests that local opioid release could occur during ACC-DBS to either engage the DPMS or suppress thalamocortical relay of noxious sensory information. Preclinical and clinical data are needed to test these hypotheses.

### Motor cortex stimulation

For more superficial brain targets, some researchers and physicians have opted for intracortical or epidural stimulation. Using this method, a craniotomy is performed, and electrodes are placed on the surface of the brain in the epidural space. Intracortical stimulation (ICS) is used for patients with chronic neuropathic pain that cannot be treated by medication and does not respond to other forms of stimulation, such as post-stroke pain (Moisset et al., 2020). For chronic pain patients, ICS is mostly performed on the surface of the motor cortex in a procedure called intracortical motor cortex stimulation (iMCS). iMCS is typically applied at stimulus frequencies between 30 and 90 Hz and requires constant, continuous stimulation *via* an implanted device for patients to continue the therapy at all times (Fontaine et al., 2009; Lefaucheur et al., 2009). The stimulus pulse amplitude is set at 80% of the amplitude necessary to elicit a motor response, but is generally imperceptible to the patient (Moisset et al., 2020).

Primary motor cortex (M1) is not particularly rich in endogenous opioid peptides or receptors. Rat M1 exhibits radiolabeled ligand binding at MORs at intermediate levels in layers I and VI, but the level of MOR expression is much less than in nearby limbic cortical areas. Ligand binding to DORs is also very low (Lewis et al., 1983). Similarly, M1 dynorphin and enkephalin immunoreactivity reveals extremely sparse expression of these endogenous opioids (Fallon and Leslie, 1986). However, because M1 stimulation is thought to activate the DPMS, endogenous opioid signaling in downstream circuits could still be an important mechanism of action. In the rat, iMCS has been shown to effectively activate M1 layer V output neurons *via* transsynaptic mechanisms, underscoring a mechanism by which superficial electrodes can affect motor cortex output (Hussin et al., 2015). In rodents, iMCS activates PAG and decreases activity in the DH, as assessed by recordings of neuronal activity and immunohistochemistry for immediate early genes, such as cFOS (Pagano et al., 2012; França et al., 2013). Some of the strongest evidence implicating endogenous opioid signaling in M1 stimulation-driven analgesia arises from the finding in rats that the resulting analgesia is consistently blocked by systemic naloxone (Fonoff et al., 2009). Further, PAG naloxone pretreatment in rats blocked the inhibition of sensory evoked potentials in the somatosensory cortex induced by M1 stimulation (Chiou et al., 2013). These preclinical data suggest that release of endogenous opioids may be a key component of iMCS-induced analgesia.

Exactly how M1 stimulation activates the DPMS remains unclear. In rats, iMCS activates striatum, cerebellum and some thalamic areas, while responses to noxious stimuli in VPL, S1, and PFC are inhibited (Jiang et al., 2014; Kim et al., 2016). In humans, functional imaging and electrophysiological studies have revealed that iMCS rapidly activates lateral thalamus. Hours later, activation of medial thalamus, ACC, orbitofrontal cortex (OFC), and PAG is observed. The PAG receives input from ACC and OFC, and functional connectivity between ACC and PAG in particular is associated with pain suppression in the contexts of opioid analgesia, placebo analgesia, and attentional analgesia (García-Larrea et al., 1999; Peyron et al., 2007). It is plausible to hypothesize that the prefrontal pain modulatory network engages the PAG, yet it remains unclear precisely how M1 stimulation recruits the prefrontal cortex and how this unfolds on such a slow timescale. The precentral gyrus in the macaque, which contains M1, additionally sends projections to PAG, suggesting a possible direct route for DPMS activation *via* iMCS (von Monakow et al., 1979).

In a meta-analysis of 14 studies that used iMCS in 210 chronic pain patients, subjective classification of outcomes yielded a positive response to iMCS in ∼55% of patients, which dropped to 45% in patients that were able to be assessed more than 1 year later. For the patients that provided visual analog scale scores of pain, their pain ratings improved by 56% after receiving the intracortical stimulation. Importantly, however, in the two studies that had internal controls for stimulation by cycling through “on” and “off” stimulation periods, patients did not show significant differences in pain outcomes between the two (Fontaine et al., 2009), suggesting the possibility that at least some aspects of iMCS pain relief result from placebo effects. Alternatively, “wash-out” effects of stimulation or induction of plasticity may also contribute to persistently reduced pain outcomes during the “off” stimulation periods. Future experiments are required to parse the contribution of these factors.

As assessed by PET imaging using [11C]diprenorphine, iMCS leads to endogenous opioid release in patients with refractory neuropathic pain in anterior midcingulate cortex (aMCC), PAG, PFC, and cerebellum, with aMCC and PAG changes correlating with pain relief (Maarrawi et al., 2007). Additionally, high opioid receptor availability in insula, thalamus, PAG, ACC, and OFC were positively correlated with later MCS pain relief efficacy (Maarrawi et al., 2013). However, another study appears to challenge the evidence pointing to endogenous opioid recruitment of the DPMS by iMCS. Although M1 stimulation increased discharge rates in LC neurons in rats experiencing neuropathic pain, lidocaine block of LC or intrathecal alpha2-adrenergic antagonists did not attenuate M1 stimulation-induced antinociception in neuropathic pain or control rats (Viisanen and Pertovaara, 2010). Continued study is needed to elucidate the exact mechanisms of endogenous opioid release during iMCS, and how it may correlate with resultant analgesia.

### Repetitive transcranial magnetic stimulation

Repetitive transcranial magnetic stimulation (rTMS) is a non-invasive neurostimulation method during which an electromagnetic coil is placed against the scalp in alignment with a target brain region. A current is passed through the coil to produce pulsatile changes in the magnetic field surrounding the coil. This magnetic field passes through the skull and into the brain, where it induces electrical currents which modulate the activity of neurons in target regions. rTMS is most commonly used to treat depression in patients who are unresponsive to or unable to tolerate medications (Speer et al., 2000). However, a systematic review of the literature concluded that rTMS is effective for central pain, peripheral nerve disorders, fibromyalgia, and migraine, and that studies using rTMS for orofacial pain, phantom limb pain, lower back pain, and complex regional pain syndrome were promising but inconclusive (Yang and Chang, 2020). Importantly, when targeted to the appropriate brain regions, the reported rTMS effects are pain-specific (Nahmias et al., 2009).

Repetitive transcranial magnetic stimulation (rTMS) treatment paradigms are widely used in the clinic and are therefore highly standardized. Typically, a patient receives rTMS for several min per session, undergoing 10s of sessions over several months. rTMS frequencies for pain treatment range between ∼0.5 and 10 Hz, with the consensus being that frequencies greater than 5 Hz are most effective (Lefaucheur et al., 2006; Moisset et al., 2015). rTMS has been extensively studied at two sites: the dlPFC, based on its accessibility and role in pain processing, and primary motor cortex (M1). M1 rTMS has been consistently reported to provide pain relief in both chronic pain patients and experimental models of pain (Lefaucheur et al., 2006; Nahmias et al., 2009; de Andrade et al., 2011; Moisset et al., 2015). Although there is some disagreement in the literature (Yoo et al., 2006), there is a general consensus that dlPFC rTMS also provides pain relief in models of experimental pain in healthy subjects (Graff-Guerrero et al., 2005; Borckardt et al., 2007; Nahmias et al., 2009; Valmunen et al., 2009; de Andrade et al., 2011; Taylor et al., 2012). While rTMS is performed contralateral to the painful site, bilateral analgesia can be evoked in humans (Nahmias et al., 2009). M1 rTMS produces bilateral analgesia in healthy patients that does not affect thermal detection thresholds, which points toward a role for diffuse descending pain modulation (Nahmias et al., 2009). rTMS provides both short-term pain relief immediately after the stimulation session, which may take 2–3 days to reach its peak, as well as long term relief that lasts for weeks to months after the end of session in contrast with the previously introduced stimulation techniques (Lefaucheur et al., 2001, 2006). Interestingly, the impact on pain affect lasts longer than on the sensory component of pain (Passard et al., 2007).

In humans, evidence for the involvement of endogenous opioids in M1 rTMS-induced analgesia has emerged from studies in healthy subjects in which naloxone blocked the rTMS-induced short-term analgesia. However, dlPFC studies by different groups reached different conclusions. A landmark study found that naloxone attenuated the analgesic effect of M1 stimulation but not dlPFC or sham rTMS (de Andrade et al., 2011), whereas another study found that naloxone blocked the analgesic effect of dlPFC rTMS (Taylor et al., 2012). A PET study using the radioligand [11C]carfentanil administered several hours after rTMS treatment of a diffuse area containing M1 and primary somatosensory cortex in healthy subjects revealed endogenous opioid release in the ipsilateral ventral striatum, mOFC, PFC, ACC, contralateral insula, superior temporal gyrus, dlPFC, and precentral gyrus, without impacting striatal D2 receptor availability (Lamusuo et al., 2017).

### Transcranial direct current stimulation

Transcranial direct current stimulation (tDCS) applies low levels of electrical current *via* small battery powered electrodes placed on the head. Although it is not currently approved by the Federal Drug Administration in the United States as its regulatory status is only “investigational,” studies on small cohorts have shown promising results for the use of tDCS in patients with fibromyalgia, spinal cord injury, and migraine (Fregni et al., 2006a,b; Dasilva et al., 2012). In other studies, however, tDCS was not effective for chronic low back pain or in spinal cord injury (O’Connell et al., 2013; Wrigley et al., 2013). Similar to iMCS and rTMS, tDCS appears most effective when applied over the motor cortex. Interestingly, PET imaging for radiolabeled opioids revealed motor cortex tDCS-driven endogenous opioid release, which reveals a possible mechanism for the measured improvements in thermal pain thresholds (DosSantos et al., 2014). Although both tDCS and placebo stimulation caused endogenous opioid release in PAG and precuneus, tDCS alone produced analgesia and additional opioid release in left PFC. Though naloxone was not administered to determine the causality of opioid signaling in the observed analgesia, these studies suggest opioidergic signaling is responsible at least in part for the tDCS-induced pain relief.

## Future outlook

### Technological innovation

#### Stimulus pulse paradigms

In recent years, there have been several innovations regarding the electrical stimulus waveforms applied by neurostimulation therapies for chronic pain. With SCS, many of these innovations apply tonic SCS at frequencies not typically utilized by conventional (i.e., 40 to 60 Hz) SCS. Kilohertz frequency SCS (KHFSCS), ultra-low frequency SCS (ULFSCS), and burst SCS all provide pain relief without producing paresthesias. KHFSCS utilizes frequencies greater than 1,000 Hz (Kapural et al., 2015), while ULFSCS applies frequencies below 0.1 Hz (Jones et al., 2021). Burst SCS employs bursts of SCS pulses at ∼40 Hz with an intraburst frequency of 500 Hz (de Ridder et al., 2013). Similar to conventional SCS, the physiological mechanisms of analgesia for each of these novel forms of SCS are unknown, presenting the same challenges to improving their design and implementation. However, these paresthesia-free SCS waveforms allow for placebo-controlled clinical studies, providing exciting new opportunities to systematically examine the effects of these new therapies in the patient’s pain experience.

In addition to new tonic SCS waveforms, new stimulus paradigms are emerging in clinical neuromodulation. Differential targeted multiplexed SCS (DTMSCS) applies two simultaneous SCS waveforms: a lower frequency 50 Hz waveform, and a higher frequency 1,200 Hz waveform (Vallejo et al., 2020). It is hypothesized that in addition to inducing conventional segmental pain inhibition, DTMSCS also affects properties of spinal glial cells (Vallejo et al., 2020). A recent innovation in DBS, Coordinated Reset DBS (CRDBS), applies precisely timed, spatially distributed stimuli to desynchronize pathological brain activity, possibly by rectifying aberrant synapses which were remodeled by disease conditions (Tass, 2003). Interestingly, CRDBS may produce long-lasting therapeutic benefit, even after the stimulus pulse is switched off (Wang J. et al., 2016). These stimulus paradigms suggest that it is critical to consider the effects of neuromodulation therapies on pre-and post-synaptic terminals and on non-neuronal cells, and that improving our scientific understanding of how the timing of exogenous electrical stimuli is integrated by neurons and synapses may allow for the evidence-based design of novel stimulus protocols which directly target the synaptic basis of pathological neural activity.

#### Closed-loop neurostimulation

A major challenge in diagnosis and treatment of chronic pain conditions is that there are no objective biomarkers of the pain experience. Most existing neurostimulation therapies apply stimulation in an “open-loop” fashion, where electrical stimuli are delivered at a constant frequency with no variation in intensity or rate. Given temporal fluctuations in severity of pain symptoms in chronic pain patients, modulating stimulation in response to changes in neural activity or behavioral biomarkers would represent an important treatment advance and may prevent tolerance by delivering stimulation only when needed and limiting unwanted side effects. Closed-loop approaches are beginning to be adopted in neurostimulation for pain, such as monitoring the amplitude of evoked compound action potentials recorded from the dorsal columns to modulate SCS pulse amplitudes. This approach was recently demonstrated to provide superior pain relief compared to open-loop SCS (Mekhail et al., 2020). Improving our understanding of how chronic pain pathogenesis and neurostimulation therapies affect the characteristics and behavior of opioidergic (and other) circuits could reveal new biomarkers with which to design closed-loop stimulation algorithms.

#### Alternate sites for neurostimulation

Continued study of the complicated matrix of brain areas involved in pain processing has revealed other targets that may provide therapeutic benefit by neurostimulation, including the insular cortex (IC). The IC can be divided along the anterior-posterior axis, with the posterior insula (pI) participating in somatosensory features of pain, whereas the anterior portion (aI) is implicated in encoding pain unpleasantness (Craig, 2002).

Low frequency electrical stimulation of the right pI elicits nociception in humans and primates with some somatotopy (Ostrowsky et al., 2002; Mazzola et al., 2009), while high frequency stimulation of pI and aI reduces pain thresholds with no obvious side effects, consistent with insular inactivation (Denis et al., 2016; Liu et al., 2021). A form of rTMS in IC has been shown to produce bilateral thermal analgesia in humans without affecting the ability to perceive innocuous thermal or vibrotactile sensations (Lenoir et al., 2018). Similarly, pI-rTMS increases thermal pain thresholds in patients with central neuropathic pain, but this did not translate to differences in relief from chronic pain and quality of life (Galhardoni et al., 2019). Although studies have not yet extended ICS to the human insula, one preclinical study in rodents suggests a potential role for low frequency intracortical pI stimulation in relief from chronic neuropathic pain. Importantly for this review, all forms of analgesia examined in this study were blocked by naloxone, clearly implicating endogenous opioid release (Komboz et al., 2022). Although opioid peptides and receptors are prominent in pI, it remains to be determined whether local opioid signaling, activation of afferents from other structures, or projections to the DPMS are involved. Innovation in the brain areas targeted by neurostimulation techniques may elucidate stimulation paradigms that provide pain relief in the absence of adverse side effects.

### Innovating clinical paradigms

#### Pharmacological adjuvants

A key challenge with electrical stimulation of any neural structure is the cellular heterogeneity of the target. Electrical stimulation is inherently non-specific; all neurons in the vicinity of the electrode are subject to modulation, which presents a challenge when the target structure is comprised of diverse neuronal subtypes which may play distinct or even opposing functional roles in neural circuits. In some cases, it may be advantageous to preferentially modulate specific subpopulations of neurons within a target structure. For example, the PAG can be subdivided into populations of glutamatergic and GABAergic neurons with subpopulations of each type projecting to the RVM to drive descending pain modulation. We hypothesize that MOR-expressing PAG-RVM projection neurons may facilitate pain, since they are inhibited by opioid analgesics. Thus, selective recruitment of the MOR-lacking PAG projection neurons using electrical stimulation may produce the most effective pain relief.

We recently demonstrated that pharmacological adjuvants can be combined with DBS to enhance its specificity (Creed et al., 2015; Creed, 2017). Pharmacological adjuvants have also been applied in preclinical (Cui et al., 1996) and clinical (Lind et al., 2004, 2007) studies of SCS, suggesting that co-application of SCS and the GABA_*B*_ receptor agonist baclofen may increase analgesia compared to the application of a single therapy alone. Currently, the combined approach of simultaneous electrical and chemical neuromodulation is not widely adopted in the clinical neuromodulation field. However, characterizing differences in ion channel or receptor expression between functional subpopulations of a target structure could identify pharmacological targets to be chemically manipulated during concurrent electrical stimulation. A dual electrical and chemical modulatory approach may allow for greater symptom control in cases where symptoms of a given disease are governed by biochemically distinct neuronal subpopulations. This approach may improve the specificity of a therapy, and thus increase efficacy while limiting off-target effects.

The advent of non-invasive, region-specific drug delivery and devices capable of delivering simultaneous electrical and chemical stimulation (Capogrosso et al., 2018) makes this an even more exciting and tractable possibility. Recently, focused ultrasound has been used to target drug release to specific sites in the brain in a non-invasive manner (Airan and Butts Pauly, 2018; Wang et al., 2018; McMahon et al., 2021). We anticipate that light-driven activation of drugs and neurotransmitters (i.e., photopharmacology) will also emerge as a viable approach that offers improved spatial and temporal precision for *in vivo* drug delivery (Banghart and Sabatini, 2012; Font et al., 2017; Hüll et al., 2018; López-Cano et al., 2021). Photopharmacology may interface particularly well with DBS and iCS, as light sources can be readily incorporated into stimulating electrodes (Royer et al., 2010; Lechasseur et al., 2011).

#### Early stimulation

Neurostimulation therapies are usually reserved for patients who are treatment refractory to every other standard of care in chronic pain conditions and for other neurological and psychiatric disorders. However, chronic pain, like other neurological and psychiatric disorders, is a disease of neural plasticity, with reorganization of neural pathways involved in pain and affective processing contributing to the persistence of pain symptoms. Recently, it has been proposed that patients receiving stocktickerSCS to manage their chronic pain would benefit from implementing the therapy earlier in disease pathogenesis (Kumar et al., 2014; Taylor et al., 2014; Lad et al., 2016; Campos et al., 2019). Along the same lines, novel DBS protocols have been shown to effectively reverse maladaptive plasticity associated with behavioral symptoms in Parkinson’s disease (Wang J. et al., 2016; Mastro et al., 2017; Spix et al., 2021) and addiction (Creed et al., 2015; Lüscher et al., 2015). Because these protocols alter plasticity in neural circuits, their therapeutic effects outlast the duration of stimulation, which is in stark contrast to classically applied tonic ∼100 Hz DBS in which motor or psychiatric symptoms reappear nearly immediately after DBS offset (Lüscher et al., 2015). An intriguing prospect would be to apply DBS in patients with pain disorders before nociceptive and affective circuitry undergo pain-induced plasticity that contributes to affective comorbidities or cognitive symptoms of chronic pain (Andrade et al., 2013). Alternatively, designing DBS protocols capable of normalizing chronic pain-induced synaptic adaptations in nociceptive processing pathways would hold enormous therapeutic promise.

#### Novel pain assessment metrics

Accurate assessment of treatment efficacy is crucial for any therapy. The success of neurostimulation therapies for chronic pain is typically defined as a ≥ 50% reduction in a patient’s overall pain, measured by the visual analog scale (VAS), verbal rating scale (VRS), or numeric rating scale (NRS). However, subjective measurements made with different scales are not always comparable (Ohnhaus and Adler, 1975; Lund et al., 2005) and may suffer from low reproducibility (van Tubergen et al., 2002). Furthermore, some have shown that the percentage of a patient cohort satisfied with SCS is disproportionately greater than the percentage of the cohort which met the ≥ 50% reduction in VAS (Sears et al., 2011). Taken together, these findings suggest that novel, holistic assessments of a patient’s pain experience may more accurately capture the efficacy of a neurostimulation therapy than a single pain rating alone. Some have suggested that dynamic pain measures, such as temporal summation and conditioned pain modulation, which are proxy measures for central sensitization and descending inhibitory tone respectively, may hold clinical value in both patient selection and assessing the efficacy of SCS (Yarnitsky et al., 2010; Campbell et al., 2015; Sankarasubramanian et al., 2019, 2021). Others have demonstrated that composite metrics which incorporate measurements of pain intensity, physical functioning, quality of life, and affect more closely represent the patient’s impression of therapeutic benefit (Pilitsis et al., 2021). These measures could provide a more accurate and reliable readout of a patient’s experience with a therapy for use during stimulator programming and as primary endpoints in clinical trials of neurostimulation therapies.

### Improving our mechanistic understanding to improve therapeutic strategies

A key limitation facing all neurostimulation therapies is that we do not understand their therapeutic mechanisms of action. Uncovering these mechanisms may allow for the evidence-based design of targeted therapies which produce robust therapeutic benefit with minimal side effects. The study of neurostimulation therapies highlights several key knowledge gaps pertaining to understanding the neural substrates of symptom management in neurological disorders.

The rationale for selecting an implant location for a neurostimulation therapy, such as ACC-DBS, is often based on historical lesioning studies (Boccard et al., 2017). However, the selection of stimulus pulse parameters is largely initially arbitrary and empirically adjusted based on subjective patient feedback. The stimulus pulse frequencies which produce therapeutic benefit in the many therapies discussed in this review are quite variable, with some therapies using pulse frequencies greater than 100 Hz, while others use pulse frequencies closer to 20 Hz. A notable finding of the involvement of endogenous opioids in preclinical SCS studies is that opioidergic analgesia during SCS may be dependent on stimulus pulse frequency. SCS applied at 60 Hz required higher doses of naloxone to abolish SCS-induced analgesia and was sensitive to a DOR antagonist, while SCS applied at 4 Hz required lower doses of naloxone to abolish analgesia and was not sensitive to a DOR antagonist (Sato et al., 2013). These data imply that the stimulus pulse frequency, putatively the rate at which axons near the stimulating electrode are conducting artificially generated APs (McIntyre et al., 2004), may affect the characteristics of neurotransmitter release from the presynaptic terminals of stimulated neurons. Future studies should examine how varying stimulus pulse frequency affects neurotransmitter release and pre-and post-synaptic receptor activation.

Many studies of neurostimulation therapies focus on the effects of stimulation on the neurons which are directly responding to the stimulus pulse. However, the resulting effects on postsynaptic networks are likely complex and intricately involved in symptom relief. Novel experimental techniques to study the activity of large networks such as *in vivo* calcium imaging (Göbel and Helmchen, 2007) and high-density electrical recordings (Jun et al., 2017; Juavinett et al., 2019; Steinmetz et al., 2021) provide the opportunity to monitor the behavior and properties of neural networks over time. These techniques could be used to observe the network response to neurostimulation therapies (Trevathan et al., 2021). Crucially, these methods also allow for the characterization of network properties across different behavioral states (Sweeney et al., 2021). Comparing network properties during both pain pathogenesis and intervention could give key insights into the development of neurological disease and reveal novel methods for targeted intervention.

## Conclusion

Neurostimulation therapies are important tools in managing intractable chronic pain. Our incomplete understanding of the mechanisms of action of such therapies precludes their improvement to maximize pain relief. In this review, we summarized the evidence that many neurostimulation therapies for pain may provide analgesia in part by modulating opioidergic circuits throughout the neuraxis. Further study is needed to understand the mechanisms by, and extent to which, neurostimulation therapies modulate these circuits. Continued study of the interactions between exogenous electric fields and neuronal and synaptic dynamics will be critical to the evidence-based design of neurostimulation therapies which specifically target mechanisms underlying neurological disease. We believe that a multidisciplinary approach combining basic neurobiological studies, innovation in clinical paradigms, and novel technology development will be key to engineering the next generation of safe and effective therapies for chronic pain.

## Author contributions

SL, RG, GL, RS, MB, and MC wrote and edited the manuscript. All authors listed have made a substantial, direct, and intellectual contribution to the work, and approved it for publication.
